# Extracellular Vesicles in Joint Disease and Therapy

**DOI:** 10.3389/fimmu.2018.02575

**Published:** 2018-11-12

**Authors:** Janneke Boere, Jos Malda, Chris H. A. van de Lest, P. René van Weeren, Marca H. M. Wauben

**Affiliations:** ^1^Department of Equine Sciences, Faculty of Veterinary Medicine, Utrecht University, Utrecht, Netherlands; ^2^Department of Biochemistry & Cell Biology, Faculty of Veterinary Medicine, Utrecht University, Utrecht, Netherlands; ^3^Department of Orthopaedics, University Medical Center Utrecht, Utrecht, Netherlands

**Keywords:** extracellular vesicle, joint, inflammation, immune suppression, cartilage, therapy, regeneration, joint homeostasis

## Abstract

The use of extracellular vesicles (EVs) as a potential therapy is currently explored for different disease areas. When it comes to the treatment of joint diseases this approach is still in its infancy. As in joint diseases both inflammation and the associated articular tissue destruction are important factors, both the immune-suppressive and the regenerative properties of EVs are potentially advantageous characteristics for future therapy. There is, however, only limited knowledge on the basic features, such as numerical profile and function, of EVs in joint articular tissues in general and their linking medium, the synovial fluid, in particular. Further insight is urgently needed in order to appreciate the full potential of EVs and to exploit these in EV-mediated therapies. Physiologic joint homeostasis is a prerequisite for proper functioning of joints and we postulate that EVs play a key role in the regulation of joint homeostasis and hence can have an important function in re-establishing disturbed joint homeostasis, and, in parallel, in the regeneration of articular tissues. In this mini-review EVs in the joint are explained from a historical perspective in both health and disease, including the potential niche for EVs in articular tissue regeneration. Furthermore, the translational potential of equine models for human joint biology is discussed. Finally, the use of MSC-derived EVs that is recently gaining ground is highlighted and recommendations are given for further EV research in this field.

## Introduction

Joint diseases, with rheumatoid arthritis (RA) and osteoarthritis (OA) as most prevalent ones, represent a significant burden to human society, both in terms of loss of quality of life and as a significant part of total healthcare costs. Current demographic and societal developments—i.e., the rapid increase of life expectancy and the decreasing acceptance of disability—aggravate the problem quickly (1). In veterinary medicine, a similar situation exists, especially in horses, a species kept for its locomotor performance and in which joint disorders are, depending on equestrian discipline, invariably ranking first or second (after tendon injuries) as cause of lameness, and thus of disability to perform (2). Given the increasing burden joint diseases have on our society, new insights in joint biology and disease in both species can facilitate the development of novel therapies.

With respect to joint homeostasis, synovial joints can be envisaged as complex organs in which the articular tissues act as an entity: synovial membrane and cartilage stay in close contact via the synovial fluid (SF). Together, these tissues maintain the joint in a healthy steady state in physiologic conditions (3). As a consequence, imbalance in one of the tissues, due to trauma, infection or inflammation, will ultimately have impact on the entire joint (4, 5). Communication between tissues is of great importance to adequately stabilize these impaired conditions. Responses to undesirable situations are known to comprise production of catabolic cytokines, enzymes and inflammatory mediators (6), and it has been postulated that extracellular vesicles (EVs) can play a role as intercellular communication vehicle for these mediators and other biological signals in regulating immunologic processes to maintain joint homeostasis. In addition to a role during episodes of disease, EVs might also take part in the regulation of healthy joint homeostasis. There is thus an urgent need for comprehensive research of local and systemic EVs in healthy, as well as diseased joints before EV-based therapies, which could potentially assist in the resolution of inflammatory joint diseases and support repair of articular tissues, can be designed. We here outline the insights gained by recent research and the opportunities that lay ahead of us.

## EVs: complex and multi-facetted particles

**Figure 1:**
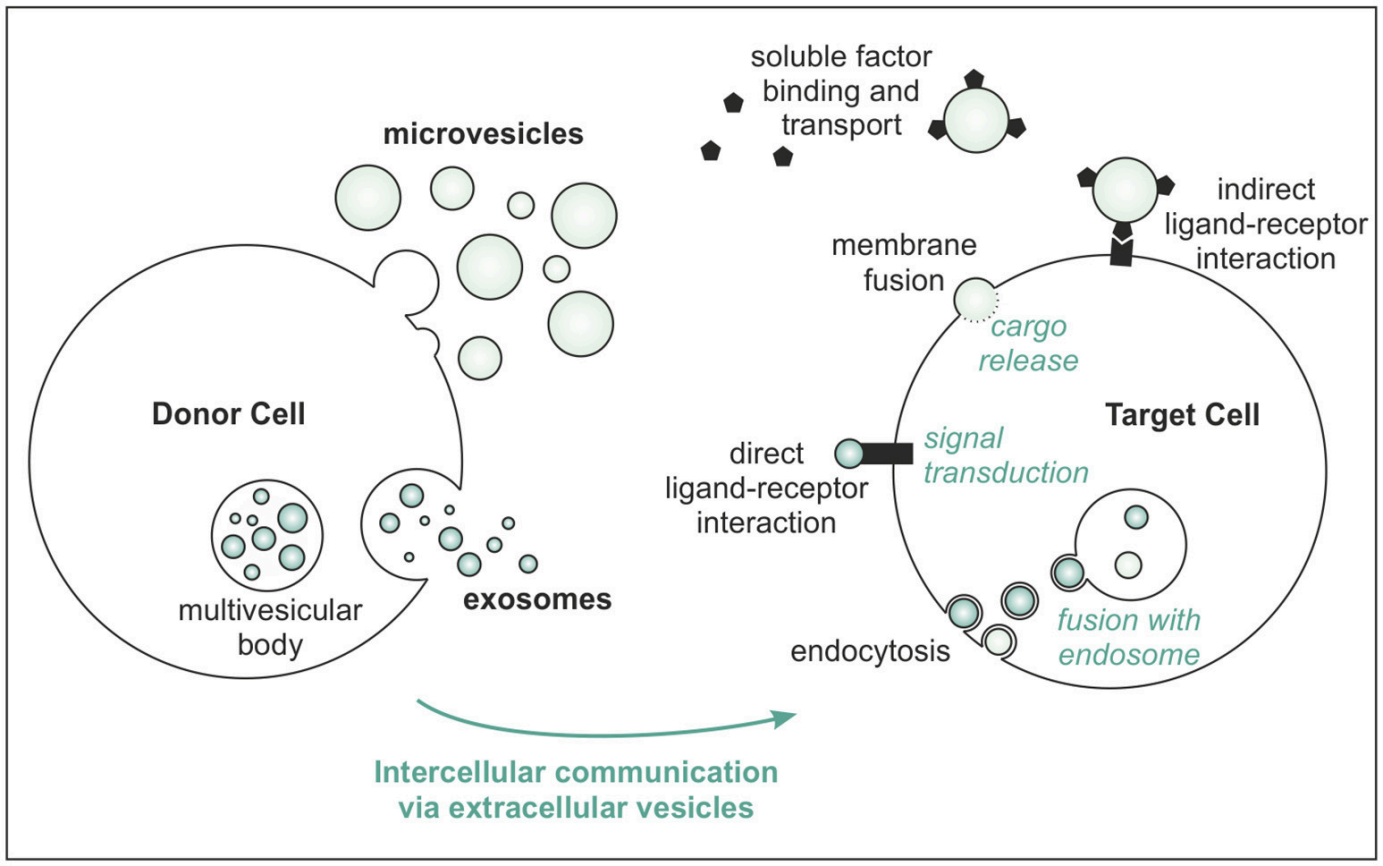
Major routes of EV biogenesis and communication with the target cell.

Extracellular vesicles are small, lipid-bilayer enclosed, cell-derived particles, specialized to facilitate cell-cell communication (7, 8). Their membrane contains proteins and lipids that mediate adherence to target cells, upon which active interaction takes place by several routes (Figure 1). In addition, soluble factors in the microenvironment of EVs can bind to their membrane and use EVs as shuttle vehicles for directed transport toward target cells (9, 10).

The major biogenesis routes of EVs follow generally one of two pathways (Figure 1): intraluminal vesicles (ILVs) can be produced within endosomes of the donor cells, resulting in multivesicular bodies (MVB). After subsequent fusion of MVBs with the plasma membrane, ILVs are released in the environment and are called “exosomes.” Alternatively, EVs originate directly from budding from the plasma membrane, referred to as “microvesicles” (7, 11). Often, the terms exosomes and microvesicles are used as equivalents for small and larger vesicles, respectively, but this classification is incorrect. Although exosomes are indeed generally smaller (as there is a limiting size for endosomal formation) and microvesicles are often detected as larger particles, both exosomes (~30–200 nm) and microvesicles (~50 nm−1 μm) can either be very small or relatively large (8). Vesicles shedding from apoptotic cells, including apoptotic bodies, are even more heterogeneous with diameters up to 5 μm.

The unique configuration of biologically active signaling molecules packaged into one small vesicle makes EVs highly efficient in bringing complex signals across (12, 13). Their lipid bilayer protects proteins and nucleic acids from the degradative extravesicular environment (i.e., the extracellular space), making stable transfer of proteins, rRNA, tRNA, miRNA, lncRNA, and (mitochondrial) DNA possible, over short and longer distances (14, 15). Also, specialized enzymes can be carried by EVs, which hence provide a tool to activate precursor molecules in the EV or in the recipient cell (13).

Extracellular vesicles typically have specific protein and lipid signatures of the cells of origin (16). In general, the EV membrane is composed of a bilayer of phospholipids, interspersed with glyco(sphingo)lipids, cholesterol, sphingomyelin, prostaglandins, integrins, tetraspanins, cell adhesion molecules and growth factor receptors (10, 15, 17). These molecules facilitate adhesion and/or fusion with recipient cells and may serve in ligand-receptor signaling. The EV membrane can also contain membrane transport proteins (e.g., sodium-dependent inorganic phosphate transporters) (18) and ion channels (e.g., annexins function as Ca^2+^ channels in matrix vesicles) (19). These characteristics enable EVs to act as sites of active processing of (signaling) molecules, in addition to being shuttling vehicles for passive transport of biologically active factors.

All cell types tested up to date can produce EVs. Production and release are tightly regulated processes which may vary between physiological and pathologic conditions (10, 20). In addition, the stimulation of cells by external stimuli can drastically change the EV-production rate and EV content or composition (21, 22). The pool of EVs found in biological fluids represents vesicles from the various cell types which are in direct contact with the fluid, or from infiltrating pathogens that also shed EVs (23, 24). In some cases, EVs can even cross epithelial and endothelial barriers, such as the blood brain barrier (25). Thus, the EV pool in body fluids reflects the systemic activity of the body as a whole or of specific organs and can be used as a monitoring tool (“liquid biopsy”) for active disease processes (26–28).

## Cartilage matrix vesicles: the ancestors of EV research

**Figure 2:**
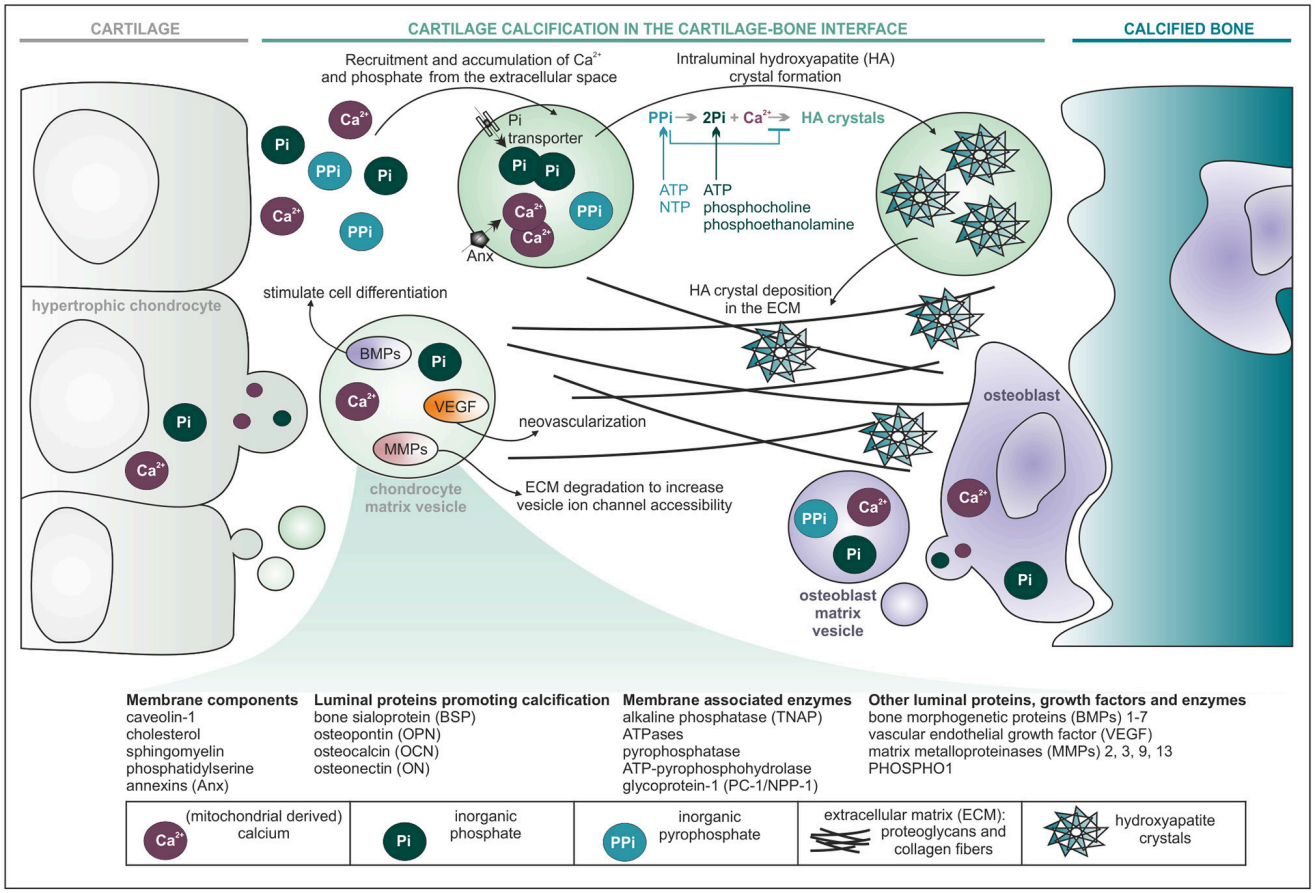
Matrix vesicles start cartilage calcification during endochondral ossification. Vesicles originating from maturing (hypertrophic) chondrocytes and osteoblasts accumulate calcium and phosphate ions in their lumen for the formation of hydroxyapatite (HA) crystals. Deposition of HA crystals in the extracellular matrix (ECM), together with calcification promoting proteins, leads to complete transformation of cartilage into bone. It is hypothesized that other factors found in matrix vesicles, such as BMPs, VEGF, and MMPs, could be involved in chondrocyte and osteoblast differentiation, neovascularisation and ECM degradation, respectively (34, 35). The electrophoretic profile of matrix vesicles is characterized by mineralisation promoting enzymes (TNAP, ATPases, etc.) that hydrolyze adenosine triphosphate (ATP) and nucleoside triphosphate (NTP) into inorganic pyrophosphate (PP_i_) and PP_i_ into inorganic phosphate (P_i_), thereby increasing concentrations of P_i_ and decreasing concentrations of PP_i_ in the vesicle lumen and its surrounding matrix (36, 37). Keeping PP_i_ concentrations low at sites of active mineralisation is critical, since PP_i_ is the most important physiologic suppressor of hydroxyapatite crystal deposition (38). To further increase the pool of P_i_ in the vesicle lumen, the enzyme PHOSPHO1 is suggested to hydrolyse luminal phosphoethanolamine and phosphocholine (derived from membrane phospholipids) in order to produce P_i_ (37, 39, 40). Active phosphate transporters in the vesicle membrane facilitate further influx of P_i_ from the ECM (18).

The discovery of matrix vesicles is historically important for the general recognition that cell-derived vesicles may be functional, instead of only representing cell debris. Matrix vesicles, for the first time described in independent parallel research by Bonucci and Anderson in 1967 (29–31) are a specialized type of EVs with diameters of 30–500 nm, known for their function in endochondral ossification, the process during which fetal growth cartilage is converted into bone. This process takes place in the hypertrophic zone of the epiphyseal growth plates and in the ossification front under the articular surface. Here, matrix vesicles are formed by budding from the plasma membrane of maturing chondrocytes and osteoblasts. These vesicles subsequently collect calcium and phosphorus in their lumina and increase the concentration of phosphate through the action of alkaline phosphatase, which facilitates mineralization of the tissue (32, 33) (Figure 2). Very uniquely, this process takes place in a polarized fashion: vesicles pinch off from the lateral side of growth plate chondrocytes and from the osteoid-facing surface of osteoblasts, in the longitudinal direction of the bone (30, 36).

The cues for the very precisely timed matrix vesicle biogenesis during endochondral ossification have not yet been completely elucidated, but an increase in intracellular Ca^2+^ concentration has been shown to induce matrix vesicle production in growth plate-derived chondrocytes *in vitro* (41). Furthermore, matrix vesicle production has been suggested to be the result of a specific form of programmed cell death by which hypertrophic chondrocytes are cleared from the growth plate and replaced by osteoblasts, leading to events of vesiculation (42).

Dysregulation of this matrix vesicle induced calcification of tissue is a feature of several joint diseases (43). In OA for example, prematurely differentiated chondrocytes are thought to release increased amounts of alkaline phosphatase and BMP-loaded matrix vesicles into the ECM, which may stimulate formation of osteosclerosis in the subchondral bone and osteophyte formation (33).

In addition to bone matrix vesicles, it is highly likely that other EV types play a role in the development of the musculoskeletal system, although direct evidence is lacking thus far. Skeletogenesis and synovial joint formation are highly orchestrated processes regulated by at least two important signaling pathways, Wnt and Hedgehog (44). These pathways steer chondrogenesis, osteoblast development and angiogenesis in concert with other regulatory factors that are expressed in the developing cartilage and perichondrium, such as BMPs, fibroblast growth factors (FGFs), TGFβ, and VEGF (45–47). These factors also have a role in homeostasis of the mature joint and all of them have been related to EVs, or found to be involved in (the regulation of) EV production and function (48, 49). It is, for example, known for Wnt signaling molecules that these are expressed on EVs derived from both Drosophila and human cells (50), indicating an evolutionary conserved process. The same holds true for Notch signaling. Notch modulates endochondral ossification (51), is required for articular cartilage and joint maintenance (52), and has been reported in multiple studies to be regulated in an EV-dependent manner (53, 54). The investigation of the role of these EVs during joint development is hence a new interesting avenue for joint biology research with potential benefits for regenerative medicine of the joint.

## EVs: regulators in inflammatory joint disease?

So far, the knowledge about SF-derived EVs and their role in articular (patho)physiology is limited to a number of descriptive investigations that have revealed the presence of EVs in SF and a small number of elegant studies pointing out specific characteristics of EVs in human joint disease (55–72).

Joint diseases are in most cases associated with (chronic) inflammation (73). In addition to the high concentrations of cytokines, chemokines, catabolic enzymes and inflammatory mediators that can be measured in the SF (74), also EVs are present in substantial amounts in SF of patients with RA and OA (55, 56, 63, 75, 76). So far, in these samples EVs have been detected that originated from synovial fibroblasts (77), platelets (60), erythrocytes (55), neutrophils (64), monocytes and T-cells (63, 78). Apart from being produced by activated synoviocytes or by infiltrating immune cells, which are a hallmark of joint inflammation, EVs in SF can be derived from blood plasma of which SF is an ultra-filtrate. Finally, chondrocytes could also be a possible EV source, but chondrocyte-derived EVs have as far as we know not been detected in SF.

Although the exact mode of action of EVs in inflammatory joint diseases still has to be elucidated, several general mechanisms that are related to inflammation have been suggested (79, 80). These include the recognition of pathogen-derived EVs by immune cells, EV-mediated shuttling of inflammatory cytokines, lipid mediators, receptors and miRNA, and the ability of EVs to carry proteolytic enzymes that cause tissue destruction and further propagation of inflammation (8, 80–82). Also, a role is claimed for EVs in autoimmune diseases, such as RA (83, 84). Interestingly, whilst most studies so far have suggested a pro-inflammatory function for SF-derived EVs, a recent study has suggested that neutrophil-derived EVs from RA SF have a protective phenotype (64, 85). Probably the most interesting and urgent question at this moment is which role EVs take in different types of joint inflammation and at different timings during the disease process. Findings from these studies will not only bring opportunities for using EVs as potential biomarkers for early detection and categorization of joint inflammation, but also guide future development of (EV-mediated) therapeutics, targeting inflammation-inducing EV pathways or suppressing inflammation, locally in the joint or at a systemic level.

## EVs for articular tissue regeneration

Cartilage and bone destruction in joint disorders is essentially irreversible and usually worsens progressively during the course of the disease. The lack of repair capacity of mature articular cartilage is notorious and has been signaled as early as the mid-1700s by William Hunter in his famous publication on cartilage structure and cartilage diseases (86). This problem has not yet been solved and the quest for innovative strategies for cartilage repair is more intense than ever, driven by societal and demographic stressors. An important development in this quest is the introduction of scaffolds constructed from biomaterials that serve as artificial matrix for the repair of (osteo)chondral defects (87). Such scaffolds can be generated using 3-dimensional (3D)-bioprinting technology (88, 89) and they can be seeded with a combination of cells and growth factors of interest (90). A comparable approach is the use of fibrin matrices containing chondrons and mesenchymal stem cells (MSCs), which currently shows promising results in a clinical trial of patients with cartilage defects (91). It recently became clear that secreted EVs are the important driving force of the bioactive capacity of these treatments. Importantly, also the bioactivity of de-cellularized ECM-derived constructs is associated with the presence of residual EVs with regenerative effects, and ECM-derived EVs are currently considered as vehicle for the functionalisation of bioscaffolds (92).

The potential of EVs for (supporting) articular tissue regeneration has already stimulated the development of biodegradable EV-like microparticles (referred to as microspheres) for controlled delivery in the joint. For example, transforming growth factor beta-1, bone morphogenetic protein 2 and insulin-like growth factor 1 have been incorporated successfully into microspheres (93). Several of these bioactive molecules have also been found as cargo of EVs (34, 94). Hence, the use of nanovesicle-mediated delivery is expected to be more efficient than using the soluble form of the proteins, which are usually prone to fast degradation after injection. In addition since several miRNAs can support chondrogenesis and decrease inflammation, incorporation of miRNAs into artificial vesicles is an interesting option to assist in regenerative strategies. Currently, several possibilities are being explored for EV-loaded scaffolds (95–98).

## Mesenchymal stem cell EVs for joint repair

Mesenchymal stem cells are seen as a promising cellular source in cartilage tissue engineering (99). The supposed mechanism through paracrine signaling is supported by several studies showing that conditioned medium from MSCs alone (100, 101), or even only the culture medium EV-fraction (102), is sufficient to induce beneficial effects to harmed tissue or to prevent tissue damage. This indicates that soluble biomolecules and possibly EVs are the main effectors in the MSC-driven regeneration cascade—not the capacity of these cells to differentiate into several lineages, as was thought for long. In 2016 we and others suggested the potential use of MSC-derived EVs as an off-the-shelf autologous regenerative treatment for tissue repair in the joint (79) and in the years that followed the first studies on this topic have been performed and showed that MSC-derived EVs indeed were able to promote osteochondral regeneration *in vivo* (97, 103) and cartilage regeneration *in vitro* (98). Recently, also the protective effect of MSC-derived EVs against bone and cartilage degradation in OA has been demonstrated (104).

In addition to MSCs, synovial membrane-derived cells with similar pluripotent characteristics as MSCs can sometimes be detected in SF or isolated from the synovial lining and their use for treatment of cartilage defects has already shown promising results (105–108). Also, specific chondrogenic progenitor cells have been found in articular cartilage and are seen as a potentially good cell source for cartilage repair (109, 110). These cells are therefore interesting EV donors for treatment of cartilage damage. Although their natural low abundance in the joint may be not sufficient for endogenous repair of cartilage defects, *in vitro* expansion of these cells and collection of the EVs they produce would allow for intra-articular administration of high concentrations of biologically active EVs.

Finally, lasting repair of joint defects can only be successful if the disturbed joint homeostasis is targeted in parallel. When joint disease presents with auto-immunity or (chronic) inflammation, immune downregulation can be achieved by using MSC-derived EVs (104). Together with the immunotherapeutic potential of other EV types (111) from different cellular sources, EV-mediated restoration of joint homeostasis can be effectuated, which is, as said, a prerequisite for durable joint repair.

## Equine model for ORTHOPEDIC EV research

The use of animal models is under debate and efforts are made to develop alternatives by using advanced (bio)technology and *in silico* modeling. Currently, there is consensus that animal models are still necessary for various purposes, which includes setting up translational studies focussing on EVs. The choice of the animal model herein is critical. The overall role and functionality of EVs is most likely conserved in the system biology of most mammals or even vertebrates (112), albeit specific EV functions can be dependent on external cues, e.g., dietary and environmental factors (113). Larger animals are more convenient than the classic laboratory species with regard to sample size for the recovery of sufficient EVs and repeated harvesting is easier, but these larger animal species come with the disadvantage that many research tools, especially antibodies and genomic sequences, are not (yet) available.

For orthopedic research the horse is one of the optimal models because of the strong similarities between equine and human joints with respect to cartilage thickness and cellular and biochemical composition of the cartilage extracellular matrix (114). For this reason, results from fundamental studies on EVs in the equine joint can—with certain caution—be extrapolated to the human situation. Since the horse is a target species in itself with a clear clinical need for improved care for joint disorders, also equine medicine will benefit from the results of these studies.

So far, the horse has been used for studying joint biology on the EV level with the purpose of unraveling fundamental EV-mediated processes (115). Possibly in the near future, the horse can also serve as animal model for testing EV-mediated treatment of joint disease, for example by using (inducible) synovitis as a model for human arthritis (116–120) or for the assessment of repair of cartilage and bone lesions with EV-mediated therapies.

## Conclusion

The interpretation of data from the relatively few studies performed so far, both for the analysis of EVs from SF and for the testing of EV-inspired drug delivery systems and EV-mediated therapies in joint disease and articular tissue repair, is hampered by the lack of consistent and standardized isolation and processing protocols (79). This is now recognized within the EV community and the International Society for Extracellular Vesicles (ISEV), and the development of experimental guidelines and requirements for standardized sample processing and EV isolation has become a high priority area in EV-research. These activities will also fuel the elucidation of the role of EVs in articular homeostasis and pathology. From this, the step toward potential use of EVs as biomarkers and even targeting or modification of specific EVs for therapeutic applications may come into reach. Given the observed anti-inflammatory and immune modulatory activity of certain EV subsets, the application of EVs for modulation of joint inflammation may be the first EV-application in joint diseases. The potential use of EVs as (decisive) stimulators of the regeneration of articular tissues in general and of hyaline cartilage in particular seems further away, as the roles of EVs in these processes are still elusive.

## Author contributions

JB drafted the manuscript and the figures. JM, CvdL, PvW, and MW edited the manuscript and made substantial direct and intellectual contribution to the work. All authors approved the manuscript before submission.

### Conflict of interest statement

The authors declare that the research was conducted in the absence of any commercial or financial relationships that could be construed as a potential conflict of interest.
